# Genetic influences on antidepressant side effects: a *CYP2C19* gene variation and polygenic risk study in the Estonian Biobank

**DOI:** 10.1038/s41431-025-01894-x

**Published:** 2025-06-27

**Authors:** Hanna Maria Kariis, Dage Särg, Kristi Krebs, Maarja Jõeloo, Kadri Kõiv, Kairit Sirts, The Estonian Biobank Research Team, Georgi Hudjashov, Lili Milani, Health Informatics Research Team, Dage Särg, Maris Alver, Kelli Lehto, Lili Milani

**Affiliations:** 1 Estonian Genome Centre, Institute of Genomics, University of Tartu, Tartu, Estonia; 2 Institute of Computer Science, University of Tartu, Tartu, Estonia

**Keywords:** Risk factors, Genetics, Psychiatric disorders

## Abstract

Antidepressant side effects are prevalent, leading to significant treatment discontinuity among patients. A deeper understanding of the underlying mechanisms could help identify individuals at risk of side effects and improve treatment outcomes.We aim to investigate the role of genetic variation in *CYP2C19* and polygenic scores (PGS) for psychiatric and side effect-related phenotypes in experiencing antidepressant side effects.We pooled Estonian Biobank data from the Mental Health online Survey (*N* = 86,244), the Adverse Drug Events Questionnaire (*N* = 49,366) and from unstructured electronic health records using natural language processing (*N* = 206,066) covering 25 common side effects. The results were meta-analysed with previously published results from the Australian Genetics of Depression Study. Among 13,729 antidepressant users, 52.0% reported side effects. In a subgroup of 9,563 individuals taking antidepressants metabolised by CYP2C19, poor metabolisers had 49% higher odds of reporting a side effect (OR = 1.49, 95%CI = 1.09–2.04), while ultrarapid metabolisers had 17% lower odds (OR = 0.83, 95%CI = 0.70–0.99) compared to normal metabolisers. PGSs for schizophrenia and depression showed the most associations with overall and specific side effects. PGSs for higher body mass index (BMI), anxiety, and systolic blood pressure were associated with respective side effects among any antidepressant and selective serotonin reuptake inhibitor (SSRI) users. Meta-analysis confirmed robust evidence linking a higher BMI PGS and weight gain across nine antidepressants and moderate evidence linking PGS for headache with headache from sertraline. Our findings underscore the role of genetic factors in experiencing antidepressant side effects and have potential implications for personalised medicine approaches to improve antidepressant treatment outcomes.

## Introduction

Antidepressants are commonly prescribed for treating psychiatric disorders, but less than half of patients with depression respond to the first prescribed medication [1]. Furthermore, 60-75% of patients experience side effects from antidepressants, and approximately 37% discontinue treatment as a result [2]. A better understanding of the underlying factors leading to antidepressant side effects is needed to improve treatment outcomes.

Antidepressant side effects can vary substantially among individuals and are likely influenced by a combination of pharmacological, genetic, and environmental factors. Genetic factors have been estimated to account for up to 42% of the variability in antidepressant treatment response [3], however the genetic basis for antidepressant side effects remains understudied. Previous research has mostly focused on drug-metabolising enzymes, such as variations in the cytochrome P450 *CYP2C19* gene. Slower metabolism resulting from *CYP2C19* variation has been linked to increased drug concentration [4, 5], improved drug response [6–9] and side effects [6–10]. However, other studies report no link or opposing results for drug response [5, 9–15] and side effects [5, 11, 15, 16]. While CYP2C19 metabolises several of the most commonly prescribed antidepressants in Estonia, other cytochrome P450 enzymes, such as CYP1A2, CYP2C9, CYP2D6, CYP2B6, and CYP3A4 also affect the biotransformation of many antidepressants [17].

In addition to genetic variation in drug-metabolising enzymes, the polygenic risk for depression and BMI, captured by polygenic scores (PGSs), has been reported to increase antidepressant side effects [18]. The Australian Genetics of Depression Study (AGDS) is a retrospective cohort study of 20,941 depression patients and the only study to date examining PGSs in relation to antidepressant side effects [18]. PGSs are calculated for each individual as the weighted sum of risk alleles associated with a trait [19]. Unlike single genetic variants, PGSs aggregate the risk conferred by multiple genetic loci, providing a broader genetic measure that better captures complex trait liability.

Currently, only few studies have thoroughly examined both genetic variation in the key drug-metabolising enzyme CYP2C19 and the broader polygenic risk captured by PGSs. To advance knowledge in this area, we implement a multi-source approach using comprehensive genotype and phenotype data from the Estonian Biobank (EstBB). Specifically, we leverage data from three distinct EstBB data layers — self-reported data from two questionnaires and natural language processing of unstructured clinical notes from electronic health records (EHRs). Although many antidepressant side effects are dose-dependent [20], information on antidepressant dose and the timing of side effect occurrence was not available in our data sources. Recognising that side effects are often underreported [21], self-reported questionnaires help capture subjective and milder side effects, while text-mining from EHRs enables the identification of more severe or long-term side effects. Integrating these diverse data sources can offer a more complete characterisation of antidepressant side effect heterogeneity [22] and improve validity by including more controls that may be underrepresented in clinical settings or targeted questionnaires. Our approach extends beyond prior studies by including both single nucleotide and structural variants in *CYP2C19* and PGSs for psychiatric and side effect-related traits, offering a broader perspective on genetic contributions. We additionally conduct the first meta-analysis on the associations between polygenic scores and antidepressant side effects, incorporating results from the AGDS [18]. By combining phenotypes from three data layers with a wide array of genetic factors, we aim to uncover novel insights into the mechanisms underlying antidepressant side effects, ultimately advancing personalised and effective treatment strategies.

## Methods

### Sample

**Fig. 1:**
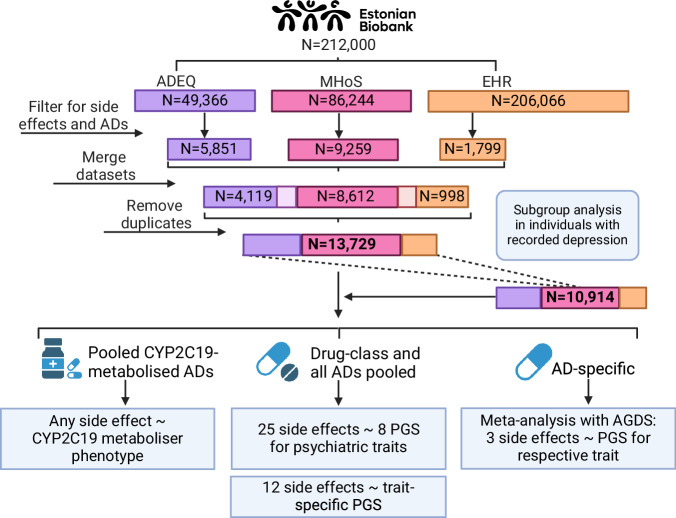
Study design overview. Three data layers containing side effect information for the Estonian Biobank participants were used. The data layers ADEQ, MHoS, and EHR were merged and antidepressants users with distinct side effects and no side effects were retained. The sample was used to carry out analyses in different drug subgroups: 1) four drugs metabolised by CYP2C19 pooled for pharmacogenetic analysis; 2) all drugs combined and drug classes separately (SSRI; SNRI+Atyp.; TCA) for PGS analyses for psychiatric trait and trait-specific analyses; 3) meta-analysis with AGDS on individual drug-level. A subgroup analysis was carried out by restricting the sample to individuals with a recorded depression diagnosis. AD—antidepressant; PGS—polygenic score; MHoS—Estonian Biobank Mental Health online Survey; ADEQ—Estonian Biobank Adverse Drug Events Questionnaire (ADEQ); EHR—electronic health records; AGDS—Australian Genetics of Depression Study; TCA—tricyclic antidepressant; SSRI—selective serotonin reuptake inhibitor; SNRI+Atyp.—serotonin-norepinephrine reuptake inhibitor and atypical antidepressant. Created in BioRender. Kariis, H. (2024) https://BioRender.com/t02y173.

The sample is derived from the volunteer-based EstBB, which has approximately 212,000 participants with genotype, health record, and questionnaire data. The EstBB represents over 20% of the adult population in Estonia [23]. We leveraged self-reported data from the Estonian Biobank Adverse Drug Events Questionnaire (ADEQ) (*N* = 49,366) and the Estonian Biobank Mental Health online Survey (MHoS) (*N* = 86,244) [24] (Fig. 1) which included questions about side effects from medications (Supplementary methods). Participants were included if they had taken at least one of the 16 most prescribed antidepressants and had reported the presence or absence of a side effect to an antidepressant. We considered 23 side effects reported by at least 100 individuals, including: sleepiness, mouth dryness, constipation, headache, weight gain, heart palpitations, sexual dysfunction, nausea, weight loss, blood pressure increase, blood pressure decrease, insomnia, agitation, allergic reaction, diarrhoea, sweating, mood change, irritability, dizziness, grogginess, anxiety, rash, and chills. The data lacked information on the timing of side effects, hence the analyses could not be adjusted for the dose of antidepressant treatment or the use of contraindicated drugs.

Additionally, we extracted antidepressant side effects from unstructured EHRs of 206,066 EstBB participants using natural language processing. Side effects were extracted using a rule-based approach and manually verified by medical experts. Only the side effects that were verified by medical experts were included in the current study (Supplementary methods). Data from three sources were merged, duplicates (*n* = 3180) removed and linked to phenotype information from data release 2023v1 (up to March 2023).

Antidepressants are prescribed for various psychiatric conditions, including major depressive disorder, chronic pain, anxiety, and sleep disorders. Accordingly, we conducted a subgroup analysis of participants prescribed at least one antidepressant for depression (International Statistical Classification of Diseases 10th Revision, ICD-10, codes F32*, F33*, F41.2) between 2004 and March 2023, as recorded in their EHRs (Supplementary Fig. 1, Supplementary Table 1). This approach aimed to reduce heterogeneity introduced by varying diagnoses, treatment, or symptomology and thus estimate whether the observed associations remained consistent within a more homogeneous population.

We additionally carried out a subgroup analysis of participants who took antidepressants metabolised by CYP2C19 since CYP2C19 affects the exposure of the antidepressant and in turn the side effects. Cases were defined as individuals reporting a specific side effect for a drug or drug class, while controls were antidepressant users without that specific side effect. The co-occurrence of side effects among cases is illustrated in Supplementary Fig. 2.

### Determining CYP2C19 metaboliser phenotypes

Star alleles were called based on phased and imputed genotypes as well as the copy number variation (CNV) data called with PennCNV (Supplementary methods). Star alleles were determined using PharmCAT 2.8.2 and an in-house pipeline, retaining only overlapping calls for downstream analyses (Supplementary methods). We included tier 1-2 *CYP2C19* star alleles and the partial deletion of CYP2C19*37. In the EstBB, CYP2C19*37 carriers were defined as the carriers of ~61.8 kbp deletions overlapping *CYP2C19* exons 1-5. Individuals were divided into five metaboliser phenotypes based on their combination of *CYP2C19* star alleles and the presence of the CYP2C19*37 partial deletion: normal (*1/*1, *1/*38, *38/*38), rapid (*1/*17, *17/*38), ultrarapid (*17/*17), intermediate (carrier of a deficient allele: *2,*3,*4,*8, *37), and poor (compound heterozygote or homozygote for deficient alleles).

### CYP2C19 variation analysis

The primary analysis focused on individuals who had taken any of the four CYP2C19-metabolised antidepressants: escitalopram, citalopram, sertraline, or amitriptyline. We investigated the reporting of any side effect, since a metaboliser phenotype causing higher exposure to a drug is typically not expected to cause a specific side effect. Logistic regression was used to test the association between side effects and the CYP2C19 metaboliser status (categorical). Normal metaboliser phenotype was set as reference. Birth year, sex, and the first 10 genotype principal components (PCs) were included as covariates. Drug-specific analyses were also conducted to assess individual drug effects.

### Polygenic scores

The imputed genotype data was used to calculate PGSs as a proxy for an individual’s genetic liability to a trait. The PGSs were based on the most recent genome-wide association study (GWAS) for each trait (Supplementary Table 2). In total, we created 19 PGS which were computed using a Bayesian polygenic prediction method, PRS-CS software (Supplementary methods). Correlations between PGSs using Pearson correlation are shown in Supplementary Fig. 3.

### The PGS analyses

The PGS analyses consisted of two sub-analyses: psychiatric trait and trait-specific analyses. The psychiatric trait analysis investigated the relationship between 25 antidepressant side effects and 8 psychiatric traits, while the trait-specific analysis focused on 12 side effects with corresponding PGS predictors.

The analyses were carried out across the following drug classes based on the anatomical therapeutic chemical classification (ATC): i) selective serotonin-reuptake inhibitors (SSRIs, ATC: N06AB), including escitalopram, citalopram, sertraline, fluoxetine, and paroxetine; ii) serotonin and norepinephrine-reuptake inhibitors, including duloxetine and venlafaxine, combined with atypical antidepressants mirtazapine, bupropion, agomelatine, vortioxetine, trazodone, and tianeptine (SNRI & Atyp. ATC: N06AX); iii) tricyclic antidepressants (TCAs, ATC: N06AA) including amitriptyline, clomipramine, nortriptyline; and iv) all antidepressants combined. The PGS analyses were conducted for all antidepressants combined to analyse side effects that would otherwise contain less than 100 cases when analysed by drug class alone.

In the psychiatric trait PGS analyses, we included 25 outcome variables, with 23 individual side effects, all side effects pooled as “any side effect,” as well as heart palpitations, weight gain, and increased blood pressure combined as “cardiometabolic side effects”. Trait-specific PGSs were tested for the following 12 side effects: sleepiness, constipation, diarrhoea, headache, weight gain, palpitations, nausea, weight loss, blood pressure increase, blood pressure decrease, insomnia, and anxiety.

Logistic regression analyses were used for testing associations between side effects and psychiatric or trait-specific PGS predictors (continuous), adjusting for birth year, sex, and the first 10 PCs. We additionally carried out these analyses on a restricted subset of participants taking antidepressants metabolised by CYP2C19, as variation in *CYP2C19* influences drug exposure which in turn can affect side effects. Analyses were corrected for multiple testing using the Benjamini–Hochberg false discovery rate (FDR) method (threshold FDR < 0.05) [25]. Psychiatric PGS analyses were corrected for 25 side effects, 8 PGSs, and 4 drug classes, while trait-specific analyses were corrected for 12 side effects and 4 drug classes with a single PGS per model.

Additionally, a forward stepwise regression was used to determine independent PGS effects of the PGSs for psychiatric traits due to collinearity between the predictors (Supplementary Fig. 3). We selected the FDR-significant associations from logistic regression results and the PGS with the lowest *p*-value when adjusted for covariates, was added to the baseline model first, followed by the next most significant predictor in the multivariate models. The process continued until no predictors met the *p* < 0.05 threshold and the models were FDR-corrected.

### Meta-analysis

The meta-analysis included the following nine drugs separately: duloxetine, mirtazapine, escitalopram, fluoxetine, sertraline, citalopram, paroxetine, amitriptyline, and venlafaxine. We pooled our results with publicly available summary statistics from AGDS [18] which included data for headache, insomnia, and weight gain. Logistic regression was used to test associations between these traits and respective PGS separately for each drug, adjusting for birthyear, sex, and the first 10 PCs. Meta-analysis was conducted with AGDS results using the fixed effects inverse variance-weighted average method. FDR-correction was performed for three phenotypes and nine drugs.

## Results

Using questionnaire and EHR text-mining data, we identified 13,729 antidepressant users with side effect information of whom 80.2% were female and had an average age of 49.9 (SD = 14.3) years (Table 1). Most had a record of depression diagnosis (*N* = 10,914, 79.5%) (Table 1, Supplementary Table 3).

**Table 1:** Sample demographics.

Characteristic	Subcategory	Cases (%)	Controls (%)	Total (%)
Sex	Female	5932 (83.1%)	5084 (71.2%)	11016 (80.2%)
	Male	1209 (16.9%)	1504 (21.1%)	2713 (19.8%)
Age	Mean Age (years ± SD)	47.4 ± 13.9	54.7 ± 13.8	49.9 ± 14.3
Metaboliser	Poor Metaboliser	108 (2.2%)	73 (1.5%)	181 (1.9%)
phenotype	Intermediate Metaboliser	1221 (24.7%)	1137 (23.0%)	2358 (24.7%)
	Normal Metaboliser	1806 (36.5%)	1690 (34.1%)	3496 (36.6%)
	Rapid Metaboliser	1528 (30.9%)	1379 (27.9%)	2907 (30.4%)
	Ultrarapid Metaboliser	288 (5.8%)	333 (6.7%)	621 (6.5%)
Subgroup	Depression	5923 (43.1%)	4991 (32.7%)	10914 (79.5%)

Percentages for cases and controls are calculated within each subcategory based on the total number of cases or controls for the characteristic. Total percentages are based on the entire study population (*N* = 13,729), except for metaboliser phenotype where the percentages are calculated only for participants who took drugs metabolised by CYP2C19 (*N* = 9563), including escitalopram, citalopram, sertraline, and amitriptyline and had complete CNV and star allele information. Cases were defined as individuals reporting a specific side effect for a drug or drug class, while controls were antidepressant users that did not report that specific side effect.

SSRIs were the most common antidepressants (*N* = 10,539, 76.8%), followed by SNRIs and the atypical antidepressants (*N* = 5589, 40.7%), and TCAs (*N* = 1724, 12.6%) (Supplementary Table 4). Approximately half of the individuals (52.0%; *n* = 7141) reported at least one antidepressant side effect (Supplementary Fig. 4, Table 1, Supplementary Table 4) with the most reported individual side effects across all antidepressants being nausea (*N* = 2155, 15.7%), weight gain (*N* = 2099, 15.3%), and sleepiness (*N* = 2055, 15.0%) (Supplementary Table 4, Supplementary Fig. 4). The least reported side effects across all antidepressants were chills (*N* = 103, 0.8%), decreased blood pressure (*N* = 134, 1.0%), and allergic reactions (*N* = 145, 1.1%).

### CYP2C19 variation in antidepressant side effects

**Fig. 2:**
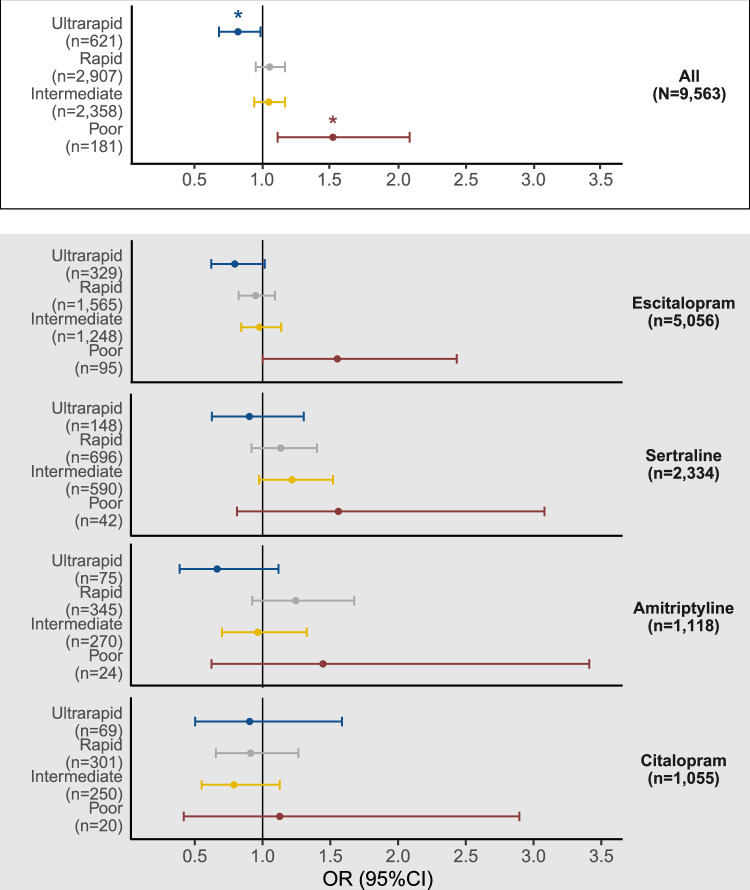
The association between CYP2C19 metaboliser status and the reporting of any side effect across escitalopram, citalopram, sertraline or amitriptyline antidepressant users (*N* = 9563). *p.value < 0.05. The normal metaboliser phenotype was used as reference. The primary analysis included all antidepressants combined; drug-specific analyses were conducted to assess individual drug effects.

The sample included 9563 individuals who had taken CYP2C19-metabolised antidepressants and had complete CNV and star allele information. The most prevalent metaboliser phenotype was normal (*N*  =  3496, 36.6%), while poor (*N* = 181, 1.9%) and ultrarapid (*N* = 621, 6.5%) phenotypes were the least common (Supplementary Table 5). Notably, the CYP2C19*37 partial gene deletion frequency in Estonia (1.5%) was ten times higher than in other European subcohorts (0.16% in the 1000 Genomes Project [26] and 0.11%, in GnomAD v4 [27] (Supplementary Table 5). Poor metabolisers were more likely to experience a side effect (OR = 1.49, 95%CI = 1.09–2.04), while ultrarapid metabolisers were less likely (OR = 0.83, 95%CI = 0.70–0.99), compared to normal metabolisers (Fig. 2, Supplementary Table 6). Drug-specific analyses were underpowered but showed consistent effect directions (Fig. 2, Supplementary Table 6).

### Psychiatric PGSs and antidepressant side effects

**Fig. 3:**
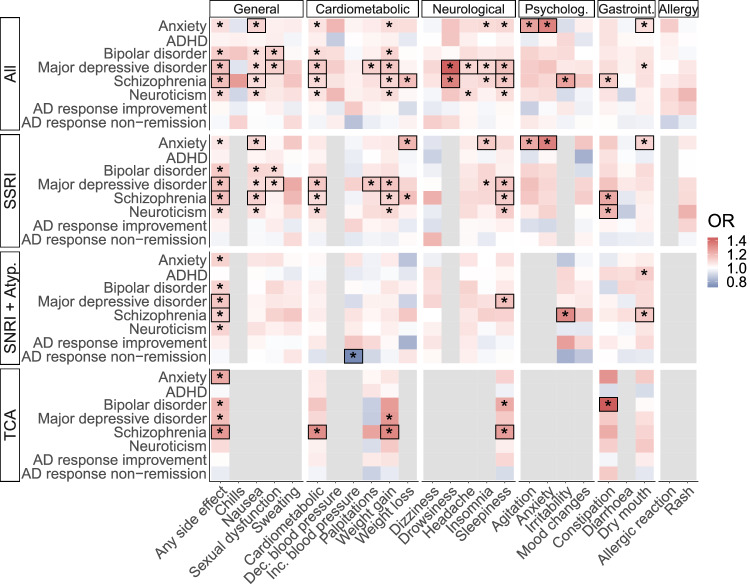
The associations between PGSs for psychiatric traits (y-axis) and side effects (x-axis) across antidepressant drug classes. Black boxes refer to independent signals in the forward regression model. AD—antidepressant; ADHD—Attention deficit/hyperactivity disorder; PGS—polygenic score; Inc.—increased; Dec.—decreased; OR—Odds ratio; Psycholog.—psychological; Gastroint.—gastrointestinal; TCA—tricyclic antidepressant; SSRI—selective serotonin reuptake inhibitor; SNRI+Atyp.—serotonin-norepinephrine reuptake inhibitor and atypical antidepressant. * FDR-corrected *p.*value < 0.05. Estimates are not reported for side effects with *N* < 100 cases for a given drug class (areas depicted in grey).

Next, we explored whether a higher genetic predisposition to psychiatric traits contributes to experiencing side effects. The analysis was conducted for all antidepressants and for each drug class, with results by drug class provided in Fig. 3 and Supplementary Tables 7-8. Most associations were observed for all antidepressants combined and SSRIs with mostly consistent findings between the two groups, likely due to the larger sample sizes and the high prevalence of SSRI users in the cohort (76.8%).

The PGS for schizophrenia (SCZ) was independently associated with experiencing side effects across all antidepressant drug classes, while the PGS for major depressive disorder (MDD) was associated with side effects in all drug classes except TCAs. The effect sizes from a forward regression model were as follows: all antidepressants pooled (SCZ PGS: OR = 1.15, 95%CI = 1.11–1.19; MDD PGS: OR = 1.14, 95%CI = 1.10–1.18), SSRIs (SCZ PGS: OR = 1.15, 95%CI = 1.10–1.19; MDD PGS: OR = 1.14, 95%CI = 1.09–1.18), SNRIs and atypical antidepressants (SCZ PGS: OR = 1.11, 95%CI = 1.04–1.17; MDD PGS: OR = 1.12, 95%CI = 1.06–1.18), and TCAs (SCZ PGS: OR = 1.26, 95%CI = 1.14–1.37). Additionally, the PGS for anxiety was linked to side effects in TCAs (OR = 1.21, 95%CI = 1.10–1.33) (Fig. 3, Supplementary Table 7-8). The associations between any side effect and PGSs for bipolar disorder (BIP) and neuroticism did not remain significant in any drug class in a forward stepwise model, indicating that their associations were likely already captured by the PGSs for MDD and SCZ due to correlation.

The PGS for SCZ and MDD similarly predicted specific side effects across all antidepressants, including drowsiness (SCZ PGS: OR = 1.27, 95%CI = 1.11–1.43; MDD PGS: OR = 1.38, 95%CI = 1.22–1.54), sleepiness (SCZ PGS: OR = 1.12, 95%CI = 1.07–1.17; MDD PGS: OR = 1.14, 95%CI = 1.09–1.19), cardiometabolic side effects (SCZ PGS: OR = 1.08; 95%CI = 1.03–1.13; MDD PGS: OR = 1.12; 95%CI = 1.07–1.16) and weight gain (SCZ PGS: OR = 1.09, 95%CI = 1.04–1.14; MDD PGS: OR = 1.13, 95%CI = 1.08–1.18) (Supplementary Table 7-8). However, the PGS for SCZ was uniquely associated with a greater likelihood of weight loss (OR = 1.17, 95%CI = 1.07–1.27), constipation (OR = 1.14, 95%CI = 1.05–1.23), irritability (OR = 1.25, 95%CI = 1.09–1.41), and nausea (OR = 1.08; 95%CI = 1.03–1.13), while the PGS for MDD was linked with a greater likelihood of heart palpitations (OR = 1.12, 95%CI = 1.06–1.19), insomnia (OR = 1.11, 95%CI = 1.04–1.17), headache (OR = 1.11, 95%CI = 1.05–1.17), and sexual dysfunction (OR = 1.10, 95%CI = 1.04–1.16). Similarly to the PGS for MDD, the PGS for BIP was positively correlated with sexual dysfunction (OR = 1.10, 95%CI = 1.04–1.16). In contrast, the PGS for anxiety (ANX) was associated with nausea (OR = 1.12, 95%CI = 1.06–1.17), agitation (OR = 1.25, 95%CI = 1.13–1.37), anxiety symptoms (OR = 1.34, 95%CI = 1.17–1.50), and dry mouth (OR = 1.09, 95%CI = 1.03–1.15) across all antidepressants.

We also observed associations in drug classes that were not present when pooling all antidepressants. Increased constipation was associated with the PGS for BIP and neuroticism, respectively in TCAs (OR = 1.44; 95%CI = 1.21–1.66) and SSRIs (OR = 1.18; 95%CI = 1.05–1.30). In SNRIs and atypical antidepressants, higher PGS for non-remission correlated with lower odds of reporting blood pressure increase (OR = 0,72; 95%CI = 0.55–0.90), while in SSRIs the PGS for anxiety was linked to weight loss (OR = 1.20; 95%CI = 1.08–1.31).

Restricting the sample to individuals with recorded depression (79.5% of the study cohort, N = 10,914) showed similar patterns (Supplementary Fig. 5, Supplementary Table 9-10). Although some associations were not statistically significant, their directions aligned with the full sample, likely reflecting reduced statistical power. The analyses on a sample restricted to CYP2C19-metabolised antidepressants showed that adjusting for the CYP2C19 metaboliser phenotype did not attenuate any of the PGS associations with side effects (Supplementary Table 11).

### Trait-specific PGS analyses

**Fig. 4:**
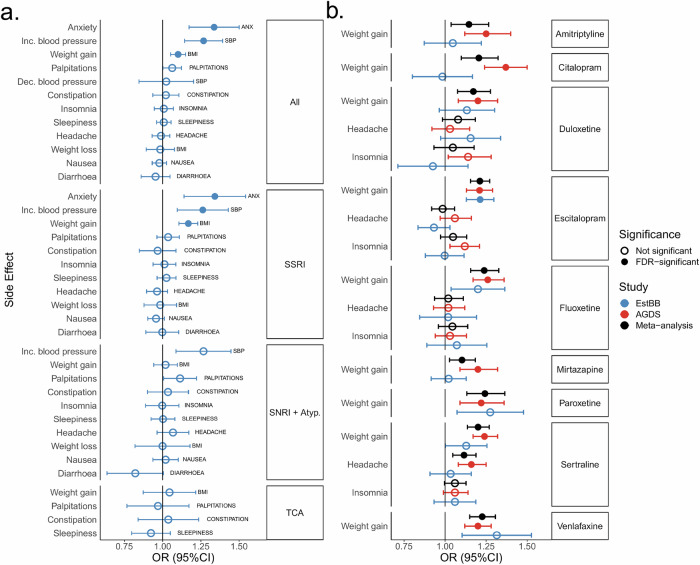
Associations between trait-specific PGSs and corresponding side effects. **a** The associations between trait-specific PGSs and side effects across antidepressant classes in the EstBB. **b** Meta-analysis results between weight gain, headache, and insomnia and their respective PGSs in EstBB and AGDS across nine antidepressants (amitriptyline, citalopram, duloxetine, escitalopram, fluoxetine, mirtazapine, paroxetine, sertraline and venlafaxine). ANX—anxiety; AGDS—Australian Genetics of Depression Study; EstBB—Estonian Biobank; OR—odds ratio; SBP—systolic blood pressure; BMI—body mass index; PGS—polygenic score; TCA—tricyclic antidepressant; SSRI—selective serotonin reuptake inhibitor; SNRI+Atyp.—serotonin-norepinephrine reuptake inhibitor and atypical antidepressant. * FDR-corrected *p*.value < 0.05.

We then investigated whether trait-specific PGSs were linked to experiencing those traits as side effects. Higher PGS for ANX was significantly linked to reporting anxiety as a side effect (OR = 1.34, 95%CI = 1.17–1.50) across all antidepressants (Fig. 4a, Supplementary Table 12). Similarly, PGSs to higher systolic blood pressure (SBP) and BMI were associated with increases in respective traits across all antidepressants (OR = 1.27, 95%CI = 1.14–1.39 and OR = 1.10, 95%CI = 1.05–1.15, respectively). Among individuals with recorded depression, the PGSs for ANX, BMI, and SBP also showed significant positive associations with their respective side effects across all antidepressants (Supplementary Fig. 6 A, Supplementary Table 13). For SSRIs, the PGSs for anxiety and weight gain remained significant, while the PGSs for SBP showed a nominal association with the trait due to its lower prevalence (*n* = 145, 1.7%) (Supplementary Fig. 6A, Supplementary Table 13). The analyses restricted to individuals taking CYP2C19-metabolised antidepressants showed that all PGS associations with side effects remained after adjusting for the CYP2C19 phenotype, compared to models without adjustment (Supplementary Table 14).

### Meta-analysis

Lastly, we conducted a meta-analysis to increase statistical power and assess the generalisability of findings between two independent cohorts. Most effect directions were consistent between the EstBB (*N* = 13,729) and AGDS (*N* = 20,941) cohorts, however in EstBB, escitalopram was the only antidepressant with a significant association between the PGS for BMI and weight gain (Fig. 4b, Supplementary Table 15). The meta-analysis identified positive associations between the PGS for BMI and weight gain across all nine examined antidepressants (Fig. 4b, Supplementary Table 15). Additionally, we identified an association between headaches and the corresponding PGS among sertraline users (OR = 1.12; 95%CI = 1.05–1.19). No significant association was observed between the PGS for insomnia and related symptoms. All significant associations persisted in individuals with recorded depression (Supplementary Fig. 6B, Supplementary Table 16).

## Discussion

This study investigated the impact of *CYP2C19* gene variation and the genetic predisposition of a comprehensive set of psychiatric and side effect-related phenotypes on the likelihood of experiencing side effects from antidepressant use. Side effects are commonly under-reported [28], hence questionnaires and text-mining of unstructured EHRs were used for a more complete picture of side effects with varying severity. We found that variation in the *CYP2C19* gene, psychiatric and trait-specific PGSs, including SCZ, MDD, ANX, BIP, BMI, SBP, and headaches, were associated with the likelihood of side effects across all antidepressants.

### CYP2C19 poor metabolisers report more side effects to antidepressants

Our study confirmed that CYP2C19 poor metabolisers have increased odds of side effects with CYP2C19-metabolised antidepressants, while ultrarapid metabolisers have lower odds of side effects. These results align with the biological mechanism that reduced enzyme activity raises drug plasma concentration and consequently side effect risk, while increased enzyme activity lowers drug concentration, reducing side effects. The results agree with studies linking lower CYP2C19 enzyme activity to higher rates of side effects [6–10] and the prescribing guidelines of the Clinical Pharmacogenetics Implementation Consortium and Dutch Pharmacogenetics Working Group which recommend dosage adjustments for certain SSRIs based on the *CYP2C19* genotype [29, 30]. Studies not finding any associations between decreased CYP2C19 enzyme activity and side effects [5, 11, 15, 16] may have lacked statistical power due to smaller sample sizes (178-1953 individuals). The larger cohort and integration of self-reported and free-text EHR data enabled more granular side effect characterisation as well as the inclusion of controls that are often under-represented in clinical records or targeted questionnaires. Additionally, including structural variants enabled more precise classification of CYP2C19 metaboliser phenotypes to detect associations, particularly considering that the CYP2C19*37 partial gene deletion frequency in Estonia is ten times higher than in other European subcohorts.

Discrepancies across studies highlight the need for further research as side effects likely result from a combination of factors, such as dosage, drug mechanisms, interactions with cytochrome P450 enzymes as well as age, sex, and nocebo effects and other non-genetic and environmental factors such as diet, lifestyle, and comorbidities [7, 18, 31, 32]. As whole-genome and long-read sequencing technologies become more advanced and widely adopted, evaluating the function of novel genetic variants will be essential to understanding individual variation in drug response. Recent approaches to predict the functional consequences of rare variants include computational algorithms trained on pharmacogenomic datasets [33, 34], such as activity prediction models, combinations of in silico and analyses [35], as well as in vivo studies through pharmacokinetic recall studies with probe-drugs [23].

### Genetic predisposition to SCZ and MDD associated with increased side effects across several drug classes

Our study extended previous research by examining links between PGSs for psychiatric traits and antidepressant side effects. We found significant independent associations between side effects and the PGSs for SCZ, MDD, ANX, and BIP across all antidepressants. PGS for SCZ and MDD were associated with higher overall side effect reporting; SCZ across all antidepressant classes and MDD across most classes. Both PGSs were also linked to specific side effects in general, cardiometabolic, and neurological symptom domains across all antidepressants. Effect sizes were generally small, however consistent in individuals diagnosed with depression and align with findings linking multiple side effects to the MDD PGS [18]. Further, adjusting for CYP2C19 metaboliser phenotype did not attenuate any associations between PGS and side effects, suggesting that CYP2C19-mediated drug exposure does not account for the observed associations. While some PGSs for psychiatric traits, such as neuroticism, showed associations with different traits, their substantial shared proportion of genetic architecture with MDD and SCZ and lower predictive power suggest that their influence on side effects may already be captured by these PGSs [36, 37]. The independent associations of the PGSs for SCZ and MDD indicate that they capture distinct molecular pathways influencing neurobiological processes and genetic predispositions related to experiencing side effects. Further investigation is required to elucidate the underlying mechanisms, particularly as they may relate to increased sensitivity to physical discomfort [38], misattributing symptoms or shared genetic mechanisms between side effects and genetic predisposition to psychiatric traits.

Importantly, this is the first study linking the PGS for SCZ to increased antidepressant side effect susceptibility. This is partly attributable to stronger predictive power driven by high heritability, polygenicity, and GWAS discoverability [39, 40]. Further, the PGS may capture more general symptoms like anxiety, rather than symptoms specific to SCZ such as hallucinations or delusions [41, 42] and thus could explain its ability to detect a greater number of associations compared to other PGSs. Since individuals diagnosed with SCZ comprised 2.5% of the sample (*n* = 341), the associations with SCZ PGS were unlikely driven by overrepresentation. Prior research has shown that MDD patients with high SCZ genetic risk often have poorer treatment outcomes, possibly due to higher symptom severity or dose requirements [13, 43, 44]. Additionally, individuals with a history of both psychosis and depression have been found to have a higher incidence of psychiatric and behavioural side effects from antiepileptic drugs [45]. Altogether, these findings suggest that individuals with a high genetic predisposition to SCZ may be more vulnerable to side effects from antidepressants and would require more careful monitoring.

The mechanisms behind these associations likely involve multiple genetic and non-genetic factors. For instance, the PGS for MDD and SCZ were both linked to several cardiometabolic side effects. The PGS for MDD was associated with weight gain and palpitations, while the PGS for SCZ was linked to weight gain and loss across all antidepressants, with mostly consistent findings in SSRIs. SSRIs are generally well tolerated, but they have been linked with weight gain [46], and citalopram specifically, to QT interval prolongation, which can lead to palpitations [47]. Therefore, while some side effects may be drug-specific, comorbidity, and shared genetic mechanisms between psychiatric and cardiovascular disease may also influence cardiometabolic side effects [48–50]. Furthermore, genetic risk for SCZ has previously been linked to worse cardiac function [48], lower BMI [50, 51], and higher BMI in patients receiving antipsychotic treatment [52]. Consequently, the cardiometabolic side effects observed may reflect an interaction of antidepressant effects, comorbid conditions, concurrent medications, and shared genetic pathways underlying psychiatric and cardiovascular traits.

These findings suggest PGSs could help identify patients at higher risk of side effects. Although not the strongest predictors [53], PGSs can be accessed early on in the treatment and can inform clinical decisions regarding drug selection or dosing, potentially enhancing drug adherence and treatment outcomes.

### Genetic predisposition to side effect-specific traits

When assessing the genetic liability to side effects specifically, we identified positive associations between anxiety, increased blood pressure, weight gain, and their respective PGSs across all antidepressants, with anxiety and SBP being novel findings. In patients with recorded depression, the associations remained significant across all antidepressants, indicating that broader genetic liability may influence side effect susceptibility beyond pharmacogenetic variants.

We compared weight gain, headache, and insomnia associations with their respective PGSs for nine antidepressants between the EstBB and AGDS [18] cohorts, finding largely consistent results. Meta-analysis with AGDS [18] revealed that the PGS for BMI was positively linked to weight gain across nine drugs, indicating that weight gain during treatment is influenced by genetic factors. Weight gain is a common treatment concern and has been linked to genetic factors in several studies [18, 52, 54]. Individuals with a high PGS for BMI have been found to gain more weight during treatment with antipsychotics [52] and antidepressants [18, 54]. This suggests that genetic predisposition to increased BMI could contribute to weight gain, irrespective of the antidepressant used. For instance, individuals with a higher PGS for BMI might experience faster weight gain. Further research could explore the molecular pathways underlying antidepressants-associated weight gain.

We additionally found that individuals with a higher genetic predisposition to headaches were more likely to report headaches when taking sertraline. Although antidepressants may be used to treat migraines, the Estonian guideline does not recommend sertraline, making it unlikely patients are prescribed it for migraine [55]. Therefore, considering an individual’s genetic predisposition to headache when prescribing sertraline and opting for alternative antidepressants may help reduce the occurrence of this side effect.

### Limitations

This study is the first to examine multiple genetic factors among antidepressant users with various psychiatric conditions. Nonetheless, there are limitations. First, self-reported side effects may be affected by recall bias and subjective interpretation. Second, cases were classified by the presence of at least one side effect per drug class, rather than drugs separately, limiting drug-specific insights. However, as most individuals reported side effects for only one drug within each class, the identified associations likely reflect drug-specific effects rather than the overlapping influence of multiple drugs. Third, PGS predictive ability depends on the trait and underlying GWAS quality. Furthermore, PGSs primarily capture common genetic variation, leaving rare and structural variants [56] unexplored. Future studies should consider additional factors, including medication dosage, lifestyle factors, comorbidities, and different ancestries to comprehensively assess genetic and non-genetic factors in antidepressant side effects.

## Conclusion

Our findings underscore the role of genetic factors in the reporting of antidepressant side effects and the value of questionnaire and EHR text-mining data in pharmacogenomic research. These results highlight the importance of genetic information in understanding the biological mechanisms of drug therapy and personalising treatment strategies.

## Supplementary information

Supplementary Information

Supplementary Tables

Supplementary Table 1

Supplementary Table 2

Supplementary Table 3

Supplementary Table 4

Supplementary Table 5

Supplementary Table 6

Supplementary Table 7

Supplementary Table 8

Supplementary Table 9

Supplementary Table 10

Supplementary Table 11

Supplementary Table 12

Supplementary Table 13

Supplementary Table 14

Supplementary Table 15

Supplementary Table 16

## Data Availability

Access to individual level data from the Estonian Biobank is regulated by the Human Genes Research Act and must be approved by the Estonian Committee of Bioethics and Human Research, see Data Access section at https://genomics.ut.ee/en/content/estonian-biobank.

## Code availability

The code used to analyse the data in this study can be obtained by contacting releases@ut.ee.
