# Supplementary material for: Genetic influences on antidepressant side effects: a CYP2C19 gene variation and polygenic risk study in the Estonian Biobank
Source: Eur J Hum Genet. 2025 Jun 27;33(10):1376–85. doi: 10.1038/s41431-025-01894-x (PMC12480030; doi:10.1038/s41431-025-01894-x)
Supplement: Supplementary file 1 — Supplementary Information [file 41431_2025_1894_MOESM1_ESM.pdf]

Supplementary Figures and Methods

Figures

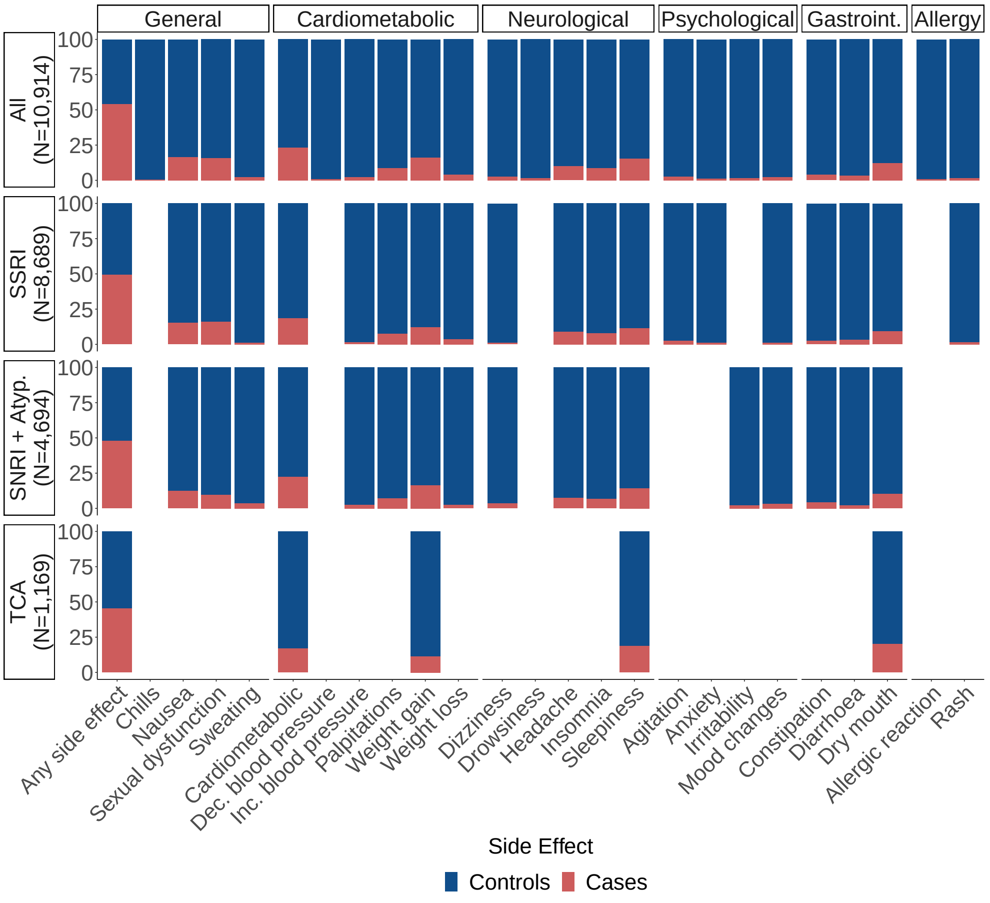

Supplementary Fig. 1. Proportion of cases reporting a side effect relative to individuals in a drug class among individuals with depression (N=10,914). Estimates are not reported for side effects with N < 100 cases for a given drug class (areas depicted in white). Inc.—increased; Dec.— decreased; Gastroint.—gastrointestinal; TCA—tricyclic antidepressant; SSRI—selective serotonin reuptake inhibitor; SNRI+Atyp.—serotonin-norepinephrine reuptake inhibitor and atypical antidepressant.

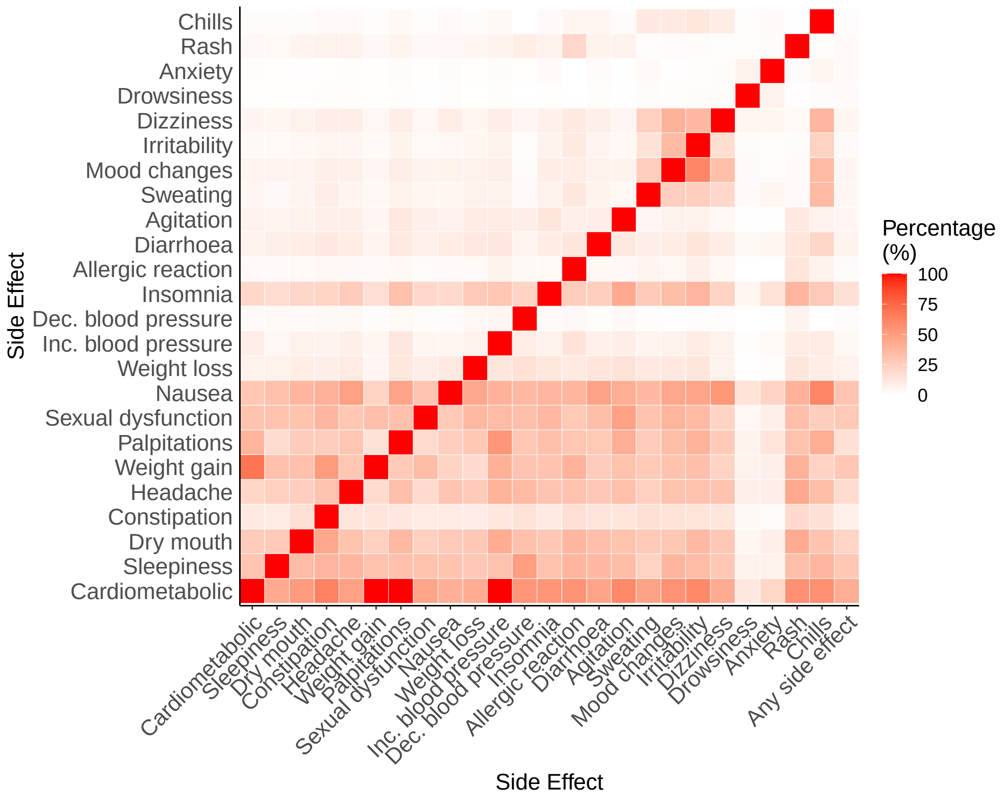

Supplementary Fig. 2. Percentage of cases reporting pairs of side effects together relative to the total occurrences of each side effect (N=13,729). Cardiometabolic side effects includes any of the following: weight gain, palpitations, blood pressure increase. Co-occurrence is measured using the cross-product of the binary matrix with the crossprod function in R. Inc.—increased; Dec.— decreased.

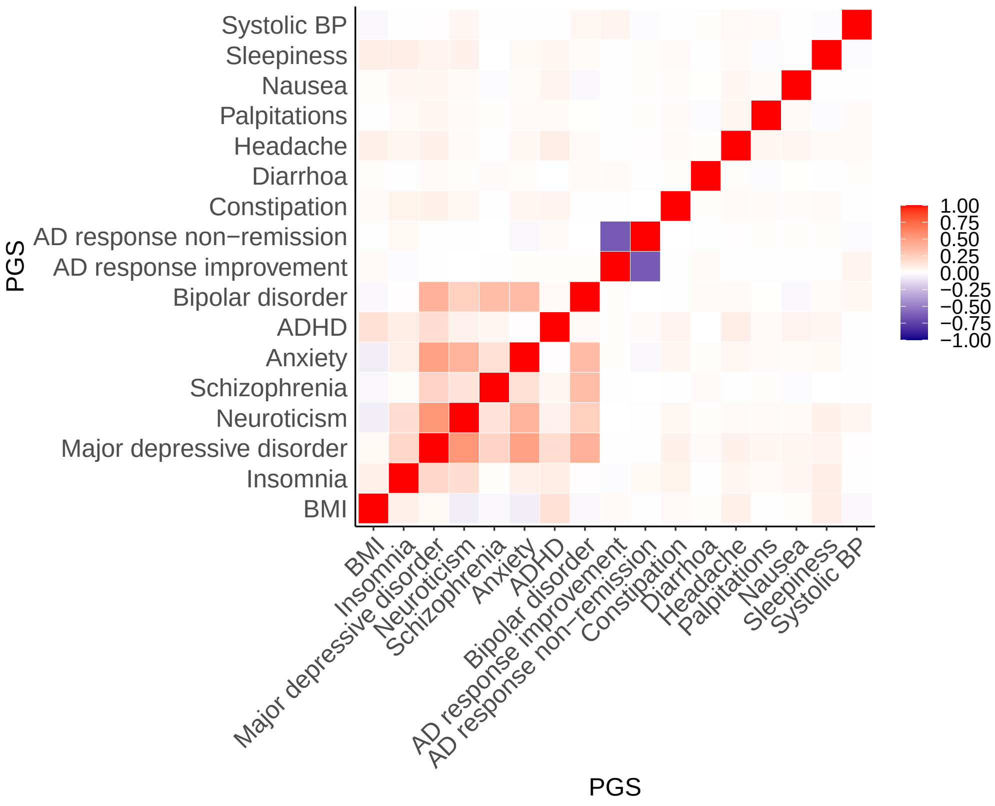

Supplementary Fig. 3. Pearson correlation between polygenic scores among 13,729 EstBB antidepressant users. AD—antidepressant; ADHD—Attention deficit/hyperactivity disorder; BP—blood pressure; BMI—body mass index; EstBB—Estonian Biobank; PGS—polygenic score.

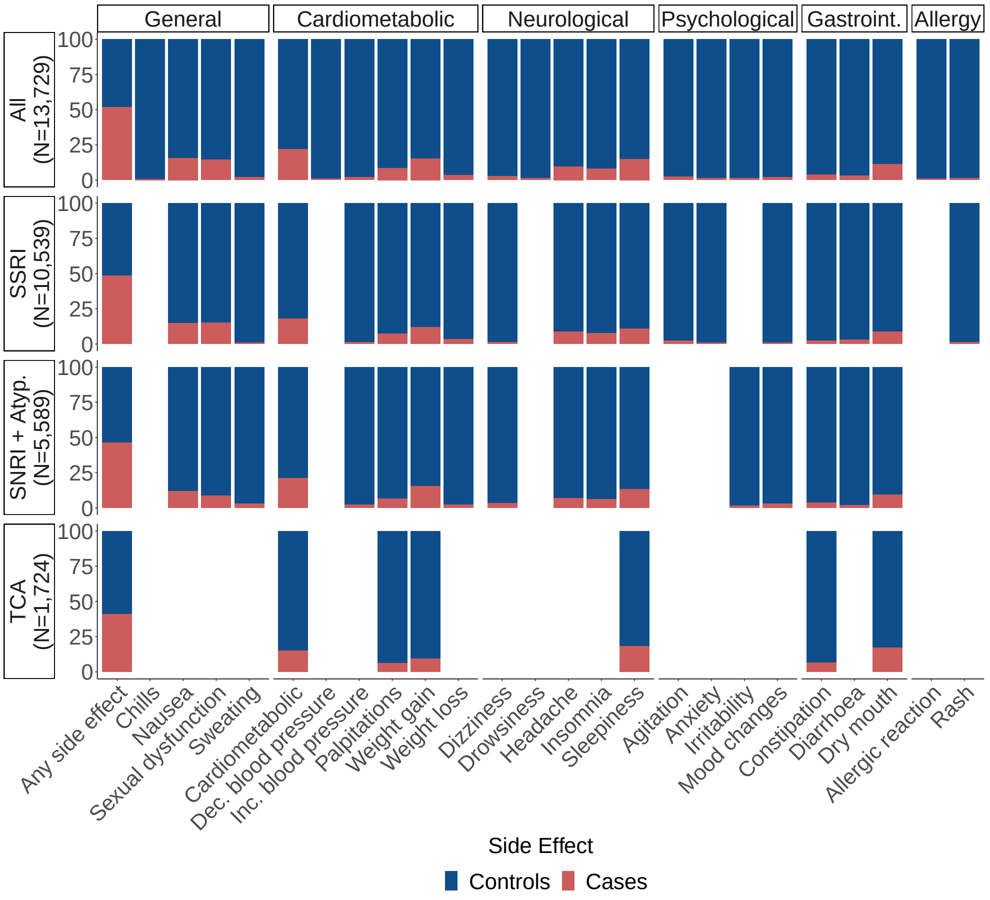

Supplementary Fig. 4. Proportion of cases reporting a side effect relative to total individuals in a drug class (N=13,729). Estimates are not reported for side effects with N < 100 cases for a given drug class (areas depicted in white). Inc.—increased; Dec.—decreased; Gastroint.—gastrointestinal; TCA—tricyclic antidepressant; SSRI—selective serotonin reuptake inhibitor; SNRI+Atyp.—serotonin-norepinephrine reuptake inhibitor and atypical antidepressant.

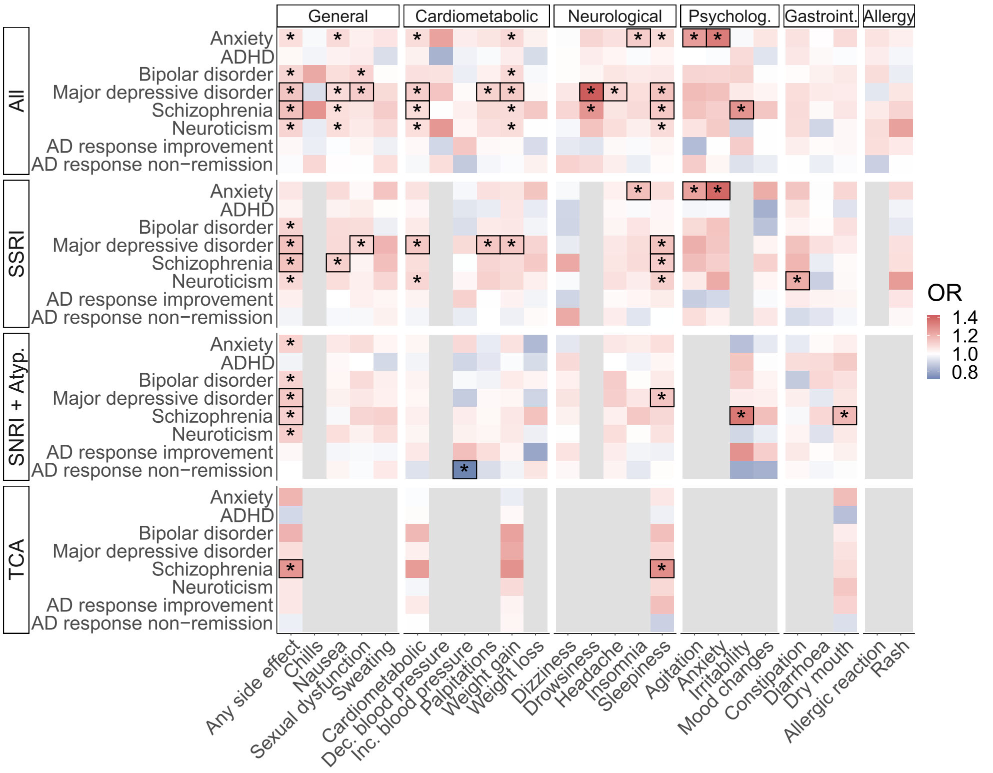

Supplementary Fig. 5. The association between PGSs for psychiatric traits (y-axis) and side effects (x-axis) across antidepressant drug classes among participants with recorded depression. Boxes refer to independent signals in a forward regression model. AD—antidepressant; PGS—polygenic score; Dec.—decreased; Inc.—increased; OR—Odds ratio; Psycholog.—psychological; Gastroint.—gastrointestinal; TCA—tricyclic antidepressant; SSRI—selective serotonin reuptake inhibitor; SNRI+Atyp.—serotonin-norepinephrine reuptake Inhibitor and atypical antidepressant. * FDR-corrected p-value < 0.05. Estimates are not reported for side effects with N < 100 cases for a given drug class (areas depicted in grey).

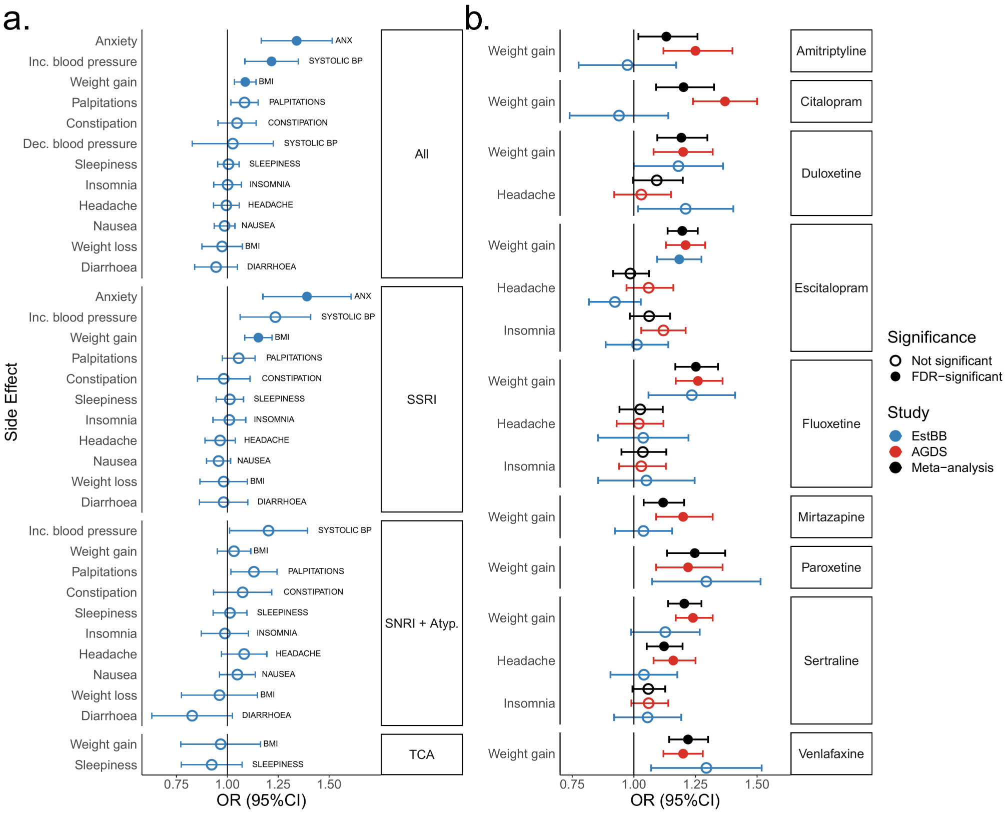

Supplementary Fig. 6. A) The association between trait-specific PGSs and side effects across antidepressant classes among participants with recorded depression. B) Meta-analysis results between weight gain, headache, and insomnia side effects and their respective PGS in EstBB and AGDS across 9 antidepressants in participants diagnosed with depression. OR—Odds ratio; ANX—anxiety; BP—blood pressure; BMI—body mass index; PGS—polygenic score; TCA—tricyclic antidepressant; SSRI—selective serotonin reuptake inhibitor; SNRI+Atyp.—serotonin-norepinephrine reuptake Inhibitor and atypical antidepressant. * FDR-corrected p.value < 0.05.

Supplementary Methods

Questionnaires

MHoS Questionnaire

The MHoS questionnaire was conducted in 2021 (March-July) among EstBB participants. Participants were first asked if their doctor had prescribed a psychotropic medication for more than two weeks for a mental health issue (responses: Yes, No, Prefer not to answer). A subsequent question requested the name of the medication from a drop-down list of common psychoactive medications, including options such as alprazolam, zopiclone, diazepam, escitalopram, betahistine, gabapentin, pregabalin, other. If the drug was not listed, there was an option to enter the name in free text. Participants were also asked to report any side effects experienced while on these medications, with a comprehensive list provided (no side effects, nausea, dry mouth, heart palpitations, constipation, diarrhoea, drowsiness, insomnia, agitation, weight gain, weight loss, increased blood pressure, decreased blood pressure, impaired sexual function, headache, itching, allergic reaction, other, prefer not to answer). Individuals were included in the study if they had reported experiencing side effects or the absence of side effect while taking an antidepressant.

ADEQ Questionnaire

The ADEQ questionnaire was conducted in 2022 (April-September) among EstBB participants. Respondents were first asked whether they had experienced side effects from any drug, with response options of Yes, No, Do not know, or Prefer not to answer. Participants were prompted to list any drugs associated with their reported side effects, referencing the Estonian Drug Registry. A checklist of potential side effects was provided, including nausea, dry mouth, palpitations, headache, dizziness, constipation, diarrhoea, drowsiness, insomnia

(disturbed sleep), agitation, weight gain, weight loss, increased blood pressure, decreased blood pressure, chills, excessive sweating, disturbed sexual function, mood changes, muscle pain, liver damage, impact on menstrual cycle, allowing participants to select all that apply. Individuals were included if they reported side effects related to at least one antidepressant, while controls were individuals who reported no side effects to any drug and had confirmed purchases of antidepressants in EHRs on at least two distinct dates.

Extraction of side effects from EHR free text

The side effect extraction from free text was performed in three stages: 1) detection of drug names and symptoms; 2) relation classification; 3) manual verification. This approach was conducted on two different occasions: first round in 2022 and the second round in 2024 as new health records had been added to EstBB.

Detection of drug names and symptoms

Both extraction rounds included the same first step: lexicon-based detection of drug names and symptoms from EHRs. To build comprehensive lexicons of drug names, we used the drug database from the Estonian Agency of Medicines. From there, we extracted all antidepressant drug names and substances (belonging to the ATC drug group N06A*, in Estonian and in Latin). When mapping them on the free text fields, we also allowed an edit distance of 1-4 depending on the length of token and the specific differences between the tokens (e.g. letters 's' and 'z' could be switched). To get a list of possible side effect symptoms, we used patient information leaflets of antidepressants which are semi-structured documents. From those, we extracted the side effect sections and split them into separate words and phrases describing side effects based on

both the structure of the document (e.g. bulleted items) and the linguistic form of the sentences/bulleted list items (e.g. side effect symptom should be a noun phrase). We lemmatized the identified side effect words and phrases using the EstNLTK toolkit and mapped them on lemmatized free text fields of the health records which had an ICD-10 diagnosis from the F group (Mental, Behavioural and Neurodevelopmental disorders)

Relation Classification and Manual Annotation

Round 1

For the first round of side effect extraction, we developed a rule-based filtering stage to reduce the number of negative examples and get an enriched sample of datapoints for manual annotation. The rules were applied on text snippets extracted from the free text fields that contained a drug name/substance and a symptom that were not farther than 200 characters from each other. At the time of the experiment, there were approximately 50,000 participants in the EstBB. In total, we retrieved 3,402 rows regarding 2,072 distinct patients that possibly had side effects from antidepressants. For manual verification, the text snippet containing the drug and symptom (marked between special characters) as well as 150 characters before the first and after the second entity were given to a human annotator for validation. The annotator manually verified 1,189 rows, containing text snippets regarding 908 patients (1,052 distinct patient-drug pairs). Out of those, we received 445 patients (520 patient-drug pairs) for whom the side effects were confirmed by the annotator.

Round 2

We included 206,066 participants with at least one epicrisis linked with the biobank during the annotation phase 2, with 49,679 of them having at least one F-diagnosis in an epicrisis. Similarly to the first annotation round, we extracted text snippets from the free text fields that contained a drug name and a reaction word/phrase not more than 200 characters apart. However, as from

annotation Round 1, we had already received a manually annotated sample of antidepressant side effects, we were able to use the same sample for fine-tuning the EstRoberta [1] pretrained text classification model for our task . For fine-tuning, we used the HuggingFace [2] library. We ran fine-tuning for 100 epochs with 500 warm-up steps, using a learning rate of 1e-5 and weight decay of 0.001. The best performance on the evaluation set was achieved at epoch 28 and consequently, the model from epoch 28 was used for AD side effect detection. Its F1-score on a hold-out test set was 0.797 with precision of 0.752 and recall 0.847. We applied the fine-tuned EstRoberta model on the retrieved 56,779 text snippets. Of those, 9,275 got the prediction from the model of belonging to the positive class, i.e. being adverse reactions between the drug and symptom under consideration. We then removed the duplicates based on patient-drug pairs (keeping only the highest confidence example for each patient-drug pair) and the data points that had already been annotated during Round 1. For manual annotation, we focused on the following drug substances: escitalopram, sertraline, citalopram, mirtazapine, and venlafaxine. In total, after cleaning and filtering, we had 2,466 rows with distinct patient-drug pairs to be manually annotated. The manual annotation process was identical to the one in Round 1. Manual verification revealed that 1,515 of those were true positive examples where the patient truly developed an adverse reaction to the drug under consideration. Combining both annotation rounds, we obtained 2,035 patient-drug pairs which were manually verified as having an adverse reaction to an antidepressant. After filtering for common side effects, we retrieved 1,858 unique patients with side effects.

Genotyping, quality control and polygenic scores

Estonian Biobank samples were genotyped at the Core Facility of Genomics, Institute of Genomics, University of Tartu, using Illumina GSAv1.0, GSAv2.0, GSAv2.0_EST, and GSAv3.0_EST arrays. Genotype data quality control was performed according to the best practices. Specifically, individuals with call rate <95%, who deviated ±3SD from the samples' heterozygosity rate mean or showed mismatch between heterozygosity of the X chromosome and sex based on phenotype data were excluded; all AT and GC SNPs, invariable SNPs, SNPs showing potential traces of batch bias, poor cluster separation results and inconsistent allele frequency among any of the EstBB genotyping experiments were removed. Pre-phasing was conducted with Eagle v2.4.1 software [3]. For imputation, the population-specific hg19 imputation reference panel of 2,695 WGS samples was used [4]. Imputation was done using Beagle 5.4 (version: 22Jul22.46e) [5].

PGSs were computed using a Bayesian polygenic prediction method, PGS-CS software [6]. PGS-CS infers posterior SNP weights using GWAS summary statistics and an external LD reference panel (1000 Genomes Project European) by placing a continuous shrinkage (CS) prior on SNP effect sizes [6]. We used the default "auto" option which uses Gibbs sampling algorithm to derive weights and excluded the HLA region for all PGSs except schizophrenia. Since schizophrenia has a highly polygenic architecture, the global shrinkage parameter in the CS prior was fixed at 1 and the HLA region was included [7, 8]. Genetic variants with minor allele frequency < 1%, imputation info score of < 0.8, significant deviation ($p < 10^{-4}$) from Hardy-Weinberg equilibrium, and ambiguous strands (A/T and C/G) were removed from PGS calculations. The PGSs were transformed into z-scores. To control for the sample overlap between discovery

and testing data, whenever possible leave-EstBB-out summary statistics were used. When leave-EstBB-out summary statistics were not available, individuals overlapping in the discovery and the testing cohorts were excluded from PGS analyses.

CYP2C19 star allele detection

The star alleles were determined using PharmCAT 2.8.2 [9] and an in-house pipeline, retaining only overlapping calls for downstream analyses [10]. We included CYP2C19 star alleles in tier 1 and tier 2 [11] and partial deletion of CYP2C19*37. Custom star alleles were excluded from the PharmCAT pipeline (https://pharmcat.org/methods/NamedAlleleMatcher-201/#exemptions). Ambiguous calls were resolved by prioritising alleles based on their functional significance in the in-house pipeline, while all possible inferred phenotypes were reported in the PharmCAT pipeline. The allele detection process was validated by comparing the identified alleles against a population-specific whole genome sequencing reference panel and known allele frequencies in European populations. Individuals were divided into five metaboliser phenotypes based on their combination of CYP2C19 star alleles and the presence of the CYP2C19*37 partial deletion: normal (*1/*1, *1/*38, *38/*38), rapid (*1/*17, *17/*38), ultrarapid (*17/*17), intermediate (carrier of a deficient allele: *2,*3,*4,*8, *37), and poor (deficient allele compound heterozygote or homozygote).

CYP2C19 partial deletion (*37) detection

We detected copy number variations (CNVs) for the genotyped EstBB cohort in 17 batches using an array-based calling software PennCNV [12]. Altogether, raw CNV calls were obtained for 220,198 samples. We excluded duplicates and 1,728 samples with inflated intensity profiles and retained raw calls for 210,213 unique samples. Finally, the sample set was filtered down to 9,628 samples who had reported to have taken drugs metabolised by CYP2C19 (amitriptyline, sertraline, escitalopram, citalopram) with side effect information. Carriers of CYP2C19*37 were defined as samples with a CYP2C19-overlapping deletion with previously established breakpoints within chr10: 96,497,325-96,559,109 (hg19) [13]. Carriers of other deletion or duplication calls overlapping CYP2C19 were flagged as ambiguous or potentially false positive and excluded from further analyses. No CYP2C19*36 full gene deletion cases were discovered. Altogether, we detected 145 (1.51%) CYP2C19*37 allele carriers and 64 (0.66%) samples with missing CNV data or ambiguous calls in our sample set. All except one CYP2C19*37 carriers were heterozygotes with respect to the deletion. For one carrier, we could not confirm the zygosity and we excluded it from further analyses. Our final dataset consisted of 9,563 individuals with 144 CYP2C19*37 carriers and 9,419 samples without CYP2C19 deletions.

References

[1] EMBEDDIA. EMBEDDIA/est-roberta; 2021. Available from: https://huggingface.co/EMBEDDIA/est-roberta.

[2] Hugging Face. Hugging Face – The AI community building the future.; 2024. Available from: https://huggingface.co/.

[3] Loh PR, Danecek P, Palamara PF, Fuchsberger C, A Reshef Y, K Finucane H, et al. Reference-based phasing using the Haplotype Reference Consortium panel. Nature Genetics. 2016 Nov;48(11):1443-8.

[4] Mitt M, Kals M, Pärn K, Gabriel SB, Lander ES, Palotie A, et al. Improved imputation accuracy of rare and low-frequency variants using population-specific high-coverage WGS-based imputation reference panel. European Journal of Human Genetics. 2017 Jul;25(7):869-76. Publisher: Nature Publishing Group. Available from: https://www.nature.com/articles/ejhg201751.

[5] Browning S, Browning B. Rapid and Accurate Haplotype Phasing and Missing-Data Inference for Whole-Genome Association Studies By Use of Localized Haplotype Clustering. American Journal of Human Genetics. 2007 Nov;81(5):1084-97. Available from: https://www.ncbi.nlm.nih.gov/pmc/articles/PMC2265661/.

[6] Ge T, Chen CY, Ni Y, Feng YCA, Smoller JW. Polygenic prediction via Bayesian regression and continuous shrinkage priors. Nature Communications. 2019 Apr;10(1):1776. Publisher: Nature Publishing Group. Available from: https://www.nature.com/articles/s41467-019-09718-5.

[7] Zheutlin AB, Dennis J, Karlsson Linnér R, Moscati A, Restrepo N, Straub P, et al. Penetrance and Pleiotropy of Polygenic Risk Scores for Schizophrenia in 106,160 Patients Across Four Health Care Systems. American Journal of Psychiatry. 2019 Oct;176(10):846-55. Publisher: American Psychiatric Publishing. Available from: https://psychiatryonline.org/doi/10.1176/appi.ajp.2019.18091085.

[8] Pardiñas AF, Kappel DB, Roberts M, Tipple F, Shitomi-Jones LM, King A, et al. Pharmacokinetics and pharmacogenomics of clozapine in

an ancestrally diverse sample: a longitudinal analysis and genome-wide association study using UK clinical monitoring data. The Lancet Psychiatry. 2023 Mar;10(3):209-19. Publisher: Elsevier. Available from: https://www.thelancet.com/journals/lanpsy/article/PIIS2215-0366(23)00002-0/fulltext#seccestitle80.

[9] Sangkuhl K, Whirl-Carrillo M, Whaley RM, Woon M, Lavertu A, Altman RB, et al. Pharmacogenomics Clinical Annotation Tool (PharmCAT). Clinical Pharmacology and Therapeutics. 2020 Jan;107(1):203-10. Available from: https://www.ncbi.nlm.nih.gov/pmc/articles/PMC6977333/.

[10] Reisberg S, Krebs K, Lepamets M, Kals M, Mägi R, Metsalu K, et al. Translating genotype data of 44,000 biobank participants into clinical pharmacogenetic recommendations: challenges and solutions. Genetics in Medicine. 2019 Jun;21(6):1345-54. Publisher: Nature Publishing Group. Available from: https://www.nature.com/articles/s41436-018-0337-5.

[11] Pratt VM, Tredici ALD, Hachad H, Ji Y, Kalman LV, Scott SA, et al. Recommendations for Clinical CYP2C19 Genotyping Allele Selection: A Report of the Association for Molecular Pathology. The Journal of Molecular Diagnostics. 2018 May;20(3):269-76. Publisher: Elsevier. Available from: https://www.jmdjournal.org/article/S1525-1578(17)30519-6/fulltext.

[12] Wang K, Li M, Hadley D, Liu R, Glessner J, Grant SFA, et al. PennCNV: an integrated hidden Markov model designed for high-resolution copy number variation detection in whole-genome SNP genotyping data. Genome Research. 2007 Nov;17(11):1665-74.

[13] Santos M, Niemi M, Hiratsuka M, Kumondai M, Ingelman-Sundberg M, Lauschke VM, et al. Novel copy-number variations in pharmacogenes contribute to interindividual differences in drug pharmacokinetics. Genetics in Medicine: Official Journal of the American College of Medical Genetics. 2018 Jun;20(6):622-9.
